# Psychological distress in newly diagnosed colorectal cancer patients following microsatellite instability testing for Lynch syndrome on the pathologist’s initiative

**DOI:** 10.1007/s10689-012-9510-1

**Published:** 2012-02-07

**Authors:** K. M. Landsbergen, J. B. Prins, H. G. Brunner, P. van Duijvendijk, F. M. Nagengast, J. H. van Krieken, M. Ligtenberg, N. Hoogerbrugge

**Affiliations:** 1 849 Department of Human Genetics, Radboud University Nijmegen Medical Centre, P.O. Box 9101, 6500 HB Nijmegen, The Netherlands; 2 Department of Medical Psychology, Radboud University Nijmegen Medical Centre, Nijmegen, The Netherlands; 3 Department of Surgery, Radboud University Nijmegen Medical Centre, Nijmegen, The Netherlands; 4 Department of Gastroenterology, Radboud University Nijmegen Medical Centre, Nijmegen, The Netherlands; 5 Department of Pathology, Radboud University Nijmegen Medical Centre, Nijmegen, The Netherlands; 6 Department of Medical Oncology, Radboud University Nijmegen Medical Centre, Nijmegen, The Netherlands

**Keywords:** Colorectal cancer, Genetic testing, Hereditary cancer, Microsatellite instability testing, Psychological distress

## Abstract

According to the Dutch Guideline on Hereditary Colorectal Cancer published in 2008, patients with recently diagnosed colorectal cancer (CRC) should undergo microsatellite instability (MSI) testing by a pathologist immediately after tumour resection if they are younger than 50 years, or if a second CRC has been diagnosed before the age of 70 years, owing to the high risk of Lynch syndrome (MIPA). The aim of the present MIPAPS study was to investigate general distress and cancer-specific distress following MSI testing. From March 2007 to September 2009, 400 patients who had been tested for MSI after newly diagnosed CRC were recruited from 30 Dutch hospitals. Levels of general distress (SCL-90) and cancer-specific distress (IES) were assessed immediately after MSI result disclosure (T1) and 6 months later (T2). Response rates were 23/77 (30%) in the MSI-positive patients and 58/323 (18%) in the MSI-negative patients. Levels of general distress and cancer-specific distress were moderate. In the MSI-positive group, 27% of the patients had high general distress at T1 versus 18% at T2 (*p* = 0.5), whereas in the MSI-negative group, these percentage were 14 and 18% (*p* = 0.6), respectively. At T1 and T2, cancer-specific distress rates in the MSI-positive group and MSI-negative group were 39 versus 27% (*p* = 0.3) and 38 versus 36% (*p* = 1.0), respectively. High levels of general distress were correlated with female gender, low social support and high perceived cancer risk. Moderate levels of distress were observed after MSI testing, similar to those found in other patients diagnosed with CRC. Immediately after result disclosure, high cancer-specific distress was observed in 40% of the MSI-positive patients.

## Introduction

Each year, more than one million patients are diagnosed with colorectal cancer (CRC) worldwide and approximately 3% have Lynch syndrome [1]. Identifying Lynch syndrome is highly relevant, because surveillance reduces morbidity and mortality in family members who carry a mutation in one of the mismatch repair genes [2]. Patients at risk for Lynch syndrome can be detected effectively with a microsatellite instability (MSI) test, which is a molecular genetic test on CRC tumour DNA [3–6]. In Lynch syndrome, almost all CRCs show high (positive) MSI.

In patients diagnosed with CRC at a relatively young age, a positive MSI test is strongly associated with genetic susceptibility [7] and can therefore be used as an indicator for Lynch syndrome. Generally, patients with an MSI-positive tumour have good overall prognoses [8, 9]. In the past, people underwent MSI testing after referral to a clinical genetic department, because of multiple CRCs in the family. However, only a minority of patients with Lynch syndrome were identified by their family history [10–13]. A new cost-effective and efficient test (MSI-testing-indicated-by-a-Pathologist (MIPA) procedure) [5, 14, 15] has enhanced the recognition of patients at risk for Lynch syndrome [5, 14, 15]. Pathologists perform MSI testing on recently diagnosed patients if they meet one of the following MIPA criteria: (1) CRC diagnosed before the age of 50 years; (2) second CRC diagnosed before the age of 70 years [5, 16, 17]. The MSI test result is reported to the surgeon. If the result is positive, the surgeon is advised to consider referring the patient for genetic counselling, which might include germline DNA analysis. One year before the introduction of the MIPA procedure, only 30% of patients at risk for Lynch syndrome were recognized as such by the traditional method based on family history [18]. Other studies also reported that family history did not adequately identify patients at risk for Lynch syndrome [10–13]. After the introduction of the MIPA procedure, performed by multidisciplinary teams that include surgeons and pathologists, the recognition of patients at risk for Lynch syndrome has increased substantially [15].

The MIPA procedure implies that CRC patients are simultaneously confronted with (1) the diagnosis of cancer and its treatment; (2) a possibly hereditary predisposition for Lynch syndrome and (3) the need to inform children and relatives about their possible cancer risks. CRC itself is known to be responsible for considerable physical and psychosocial morbidity [19]. The question therefore arises: To what extent will MSI testing add to this distress? Newly diagnosed CRC patients who were immediately offered genetic testing for hereditary CRC considered the test and the timing to be highly acceptable [20]. However, little is known about the actual psychosocial consequences of discussing a high genetic risk for Lynch syndrome with CRC patients during the treatment phase. The aim of the present study was to investigate general distress and cancer-specific distress in these patients. Social support and cancer risk perception were also studied as possible predictors of distress levels [21–24]. Furthermore, in the relatively young patients with CRC, the reactions of the partner were measured twice in the 6 months following MSI testing.

## Methods

### Patients and design

A prospective multi-centre study was performed in patients recently diagnosed with CRC to assess their psychological and cancer-specific distress and the response of their partner following MSI testing [5]. Inclusion criteria were (1) patient younger than 50 years at CRC diagnosis, or (2) second CRC diagnosed before the age of 70 years.

Psychological assessment took place using questionnaires immediately after MSI result disclosure (T1) and 6 months later (T2). Patients who had been diagnosed more than 6 months earlier were excluded. We chose a follow-up of 6 months because some patients need adjuvant therapy that can involve a treatment trajectory of 12 months or more [25]. As adjuvant therapy might also affect psychological distress levels, this variable was included in our analyses.

### Procedure

Between September 2006 and March 2007, 30 Dutch hospitals were invited to participate in the MIPAPS (Psychosocial Impact MIPA Strategy) study. Hospitals were selected based on their previous participation in the MIPA implementation study [15] and several additional hospitals were also approached in the neighbouring regions. From March 2007 to September 2009, we identified 400 patients who had been newly diagnosed with CRC and undergone an MSI test. The patient’s surgeon was requested to invite the MIPA patient and his or her partner to participate in the MIPAPS study. The majority of hospitals that took part in the study could not perform the MSI-test themselves and sent the tumour tissue sample to a specialized centre, e.g. the Department of Pathology of the Radboud University Medical Centre in Nijmegen. Once the result was available, it was sent to the pathologist, who then passed it on to the surgeon. Consequently, it was not until about some months after surgery that the surgeon could communicate the MSI test result to the patient. The time limit for inclusion by the treating physician was 6 months after CRC diagnosis. As a result of medical confidentially, we were unable to determine exactly how many patients had been invited by their surgeon and whether or not they had declined the invitation. As soon as written informed consent was received, questionnaires were sent to the patient and his or her partner. The study was approved by the Ethical Committee Arnhem-Nijmegen (CMO No. 2006/042).

### Assessments

#### Distress

The Symptom Check List-90 (SCL-90) with a 5-point Likert scale (scores 1–5) was used to assess psychopathology. A total SCL-90 score of more than 160 is indicative of high psychological distress, while a score of more than 200 is indicative of a psychiatric disorder [26, 27].

The Profile of Mood States-Short Form [28] was used to assess affective states. Items were rated on a 5-point scale (0–4) and produced scores of 0–32 for depression, 0–28 for anger, 0–24 for fatigue, 0–24 for tension and 0–20 for vigour. This questionnaire has previously been validated for cancer patients [29].

#### Cancer-specific distress

The Impact of Event Scale (IES) [30, 31] was used to assess CRC-specific distress. All 15 items were rated on a 4-point Likert scale (scores 0, 1, 3, 5). Total IES scores could range from 0 to 75. A total IES score of 9–25 is indicative of moderate adaptation difficulties, while a score ≥26 is considered to be indicative of clinical adaptation difficulties and reflect a need for [32] psychological or psychiatric support.

#### Colorectal cancer risk perception

Lifetime risk of CRC was measured with a single question from the Cancer Risk Perception List [22–24]: “My risk of having colorectal cancer again is….”. The patients marked their risk perception on a Visual Analogue Scale (VAS 0–100%). Absolute risk ranges were classified as follows: 0–20 (low); 20–40 (moderate); 40–60 (fairly high); 60–80 (high); 80–100 (very high).

#### Social support

Social support was assessed on a 4-point Likert Scale with the Dutch self-report Inventory for Social Support (ISS). The inventory comprises three scales: (1) potential emotional support: range 5–20, moderate 13–15; (2) actual emotional support: range 3–12, moderate 5–7 and (3) visits: range 2–8, moderate 5–6 [33]. Higher scores indicate greater social support.

#### Partner’s reaction to providing care and support for the cancer patient

At T1 and at T2, the patient’s partner was invited to complete two questionnaires. The effect of providing care and support for the cancer patient was measured with the *Caregiver Reaction Assessment* (CRA-D) using the 7-item subscale self-esteem. Perceived impact was rated on a 5-point Likert scale. Higher scores represented lower self-esteem [34, 35]. Perceived distress caused by the provision of informal care was measured using the validated 9-item Dutch self-report questionnaire EDIZ (one dimensional assessment of care burden) [36]. Total scores were interpreted in three categories: 9–20 (low burden), 21–32 (overburdened) and 33–45 (severely overburdened) [23, 37].

### Statistical analysis

Demographic and clinical characteristics of the MSI-positive group and MSI-negative group were analysed using the independent *T* test for the continuous variables and Pearson’s exact χ^2^ test and McNemar’s test for the categorical variables. General linear models for repeated measurements (GLM RP) were used to test for differences in psychological distress and partner’s care burden over time. Correlations between distress and demographic variables, social support and cancer risk perception were assessed by Spearman’s Rank Correlation, represented by Spearman rho (ρ). SPSS 16.0 statistical package was used to analyse the data.

## Results

### Patient characteristics

Response rates of the MSI-positive patients (MSI-high CRC) and MSI-negative patients (microsatellite stable-CRC or MSI-low CRC) were 23/77 (30%) and 58/323 (18%), respectively. No significant differences were found in age at diagnosis (*t* = 0.095; *p* = 0.8) or gender (*t* = 0.076; *p* = 0.6) between the participants and the non-responders. The participating CRC patients (n = 81) were aged 48 ± 10 years. Data were obtained 5 ± 3 months after CRC diagnosis (T1); 50% of the participants were male. Demographic and medical characteristics (T1) of the MSI-positive and MSI-negative groups are shown in Table 1. Tumour characteristics were significantly different between the two groups. As expected, more patients in the MSI-positive group had a right-sided tumour and a low TNM tumour stage. Moreover, fewer of these MSI-positive patients had received adjuvant therapy. Partner response rates in the MSI-positive and MSI-negative patients were 56% (n = 13) and 63% (n = 37), respectively (28 women, 22 men). Surgeons did not always know whether a patient had a partner or not, so these percentages were based on all the patients who participated.

**Table 1:** Baseline characteristics of the patients

	MSI-positive group^a^	MSI-negative group	
	n = 23	n = 58	*p*
*Patient characteristics*			
Age at cancer diagnosis	48 ± 10	48 ± 12	ns^b^
Male	12 (52%)	29 (50%)	ns^c^
Married or cohabiting	23 (100%)	50 (86%)	ns^c^
Having children	21 (91%)	49 (89%)	ns^c^
Educational level > high school	14 (61%)	30 (52%)	ns^c^
Religious	17 (74%)	34 (59%)	ns^c^
CRC diagnosed below 50 year	15 (65%)	38 (66%)	ns^c^
Second CRC diagnosed below 70 year	7 (32%)	20 (35%)	ns^c^
*Tumour characteristics*			
Right sided tumour location	11 (50%)	15 (26%)	0.06^c#^
TNM stage I or II	16 (73%)	26 (45%)	0.04^c*^
Adjuvant therapy	12 (55%)	40 (78%)	0.04^c*^

^a^MSI-positive means that the MSI-test in the tumor is positive and is performed at the initiative of a pathologist, either because the CRC was diagnosed below 50 years or because it was the second CRC below 70 years

^b^Independent samples *T* test

^c^Pearson chi-square test

^#^ *p* < 0.1; * *p* < 0.05; *ns* not statistically significant

### Distress

**Fig. 1:**
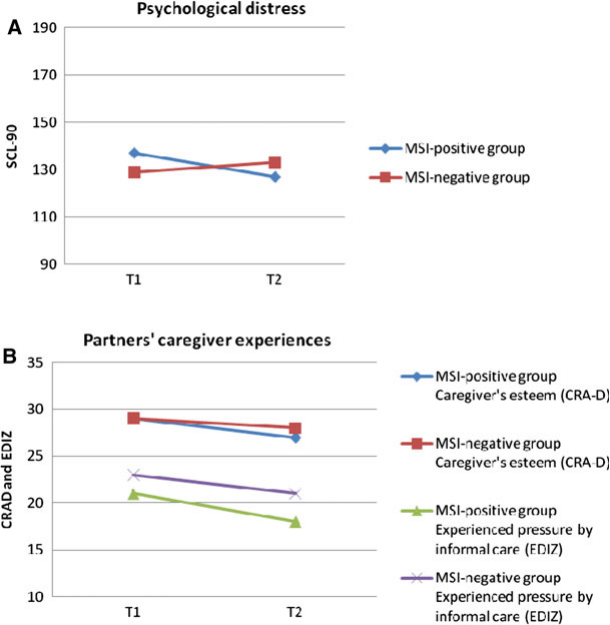
**a** Course of mean levels of psychological distress in 22 MSI-positive^ and 51 MSI-negative patients with CRC. **b** Course of mean levels of caregiver experiences in 13 partners of MSI-positive^ patients and 37 partners of MSI-negative patients with CRC, a lower CRA-D score indicates higher caregiver’s esteem, a higher EDIZ score indicates higher perceived distress by informal care. **a** SCL-90: *p* < 0.03 (interaction-effect); **b** CRAD: *p* = 0.01 (time-effect), EDIZ: *p* = 0.04 (time-effect). ^ MSI-positive means that the MSI-test in the tumour is positive and is performed at the initiative of a pathologist, either because the CRC was diagnosed below 50 years or because it was the second CRC below 70 years

At T1, psychological distress scores (SCL-90) in the MSI-positive group and MSI-negative group were within the same range (Table 2). Mean scores of psychological distress were moderate at T1 (131 ± 38) and T2 (131 ± 46), which was lower than in breast cancer patients (151 ± 45) and haematological cancer patients (145 ± 33), but similar to the scores in patients with other solid tumours (130 ± 25) [23, 38]. In the course of the study, the results of the GLM for repeated measures analysis showed differences in psychological distress between the two groups (Fig. 1a). A significant interaction effect was found between the MSI test result and the time of assessment, which indicated a decrease in psychological distress in the MSI-positive group and an increase in the MSI-negative group between T1 and T2 (F(1,71) = 4.91, *p* = 0.03). Although the differences were statistically significant, the changes in psychological distress may not be clinically relevant, because the mean distress levels did not reach the cut-off score of 160 (indicative of high distress). At T1, almost twice as many patients in the MSI-positive group reported high psychological distress (27%) than in the MSI-negative group (14%), but this difference was not statistically significant. In the MSI-positive group, percentages of patients with high general distress were 27% at T1 and 18% at T2 (McNemar test, exact *p* = 0.5); in the MSI-negative group, these percentages were 14% at T1 and 18% at T2 (McNemar test, exact *p* = 0.6). Thus, at T2, 18% of the patients in the two groups still reported high psychological distress.

**Table 2:** Psychosocial outcomes of MSI-positive (n = 22*) and MSI-negative (n = 51*) patients and their partners (n = 13 and n = 37 respectively), immediately after MSI-test disclosure (T1) and 6 months later (T2)

	MSI-positive patients^a^			MSI-negative patients		
	T1	T2	Δ	T1	T2	Δ
CRC patients						
Psychological distress^b^	137 ± 45	127 ± 51	−10 ± 27	129 ± 37	133 ± 43	4 ± 24
Cancer specific distress^c^	22 ± 22	18 ± 17	−4 ± 14	21 ± 15	22 ± 17	1 ± 13
Depression^d^	4 ± 6	3 ± 5	−1 ± 4	3 ± 4	5 ± 6	2 ± 5
Anger^d^	5 ± 6	5 ± 6	0 ± 4	3 ± 4	5 ± 6	1 ± 4
Fatigue^d^	8 ± 6	5 ± 6	−3 ± 5	6 ± 6	6 ± 6	0 ± 4
Tension^d^	5 ± 5	4 ± 5	−1 ± 3	3 ± 4	5 ± 5	1 ± 4
Vigor^d^	9 ± 4	11 ± 5	2 ± 5	9 ± 5	10 ± 5	1 ± 5
Cancer risk perception^e^	44 ± 23	53 ± 23	10 ± 23	43 ± 21	48 ± 22	5 ± 24
Social support^f^						
Potential emotional trust	16 ± 4	16 ± 4	0 ± 4	17 ± 4	16 ± 4	0 ± 3
Actual emotional trust	7 ± 2	7 ± 2	0 ± 2	7 ± 2	6 ± 2	0 ± 2
Visits	6 ± 1	6 ± 2	0 ± 1	6 ± 1	6 ± 1	0 ± 1
Partners of CRC patients						
Caregiver’s esteem^g^	29 ± 4	27 ± 5	−3 ± 5	29 ± 3	28 ± 4	0 ± 4
Perceived stress by care^h^	21 ± 4	18 ± 5	−2 ± 5	23 ± 6	21 ± 5	−1 ± 6

^a^MSI-positive means that the MSI-test in the tumor is positive and is performed at the initiative of a pathologist, either because the CRC was diagnosed below 50 years of because it was the second CRC below 70 years. Δ, difference scores (T2-T1), based on the original scores before rounding

^b^SCL-90

^c^IES-CRC

^d^POMS

^e^Life time risk to get CRC again

^f^ISB

^g^CRAD

^h^EDIZ

* Patients who filled in both questionnaires (T1 and T2)

**Fig. 2:**
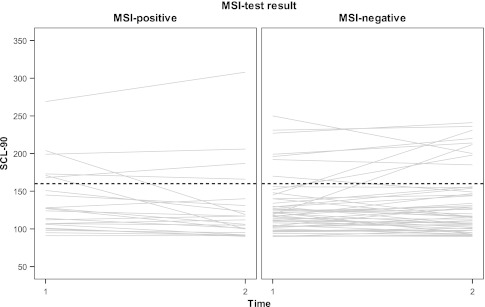
Psychological distress per MIPAPS patient at T1 and T2. A score above the cut off of 160 (*dotted line*) indicates high psychological distress

Individual psychological distress levels in the MSI-positive group and MSI-negative group are shown in Fig. 2. Per patient, psychological distress generally remained stable over time in the two groups. Psychological distress at T1 was significantly correlated with female gender (ρ = 0.269, *p* = 0.02), low social support (potential support ρ = −0.298, *p* = 0.01, visits ρ = −0.263, *p* = 0.03) and high CRC lifetime risk perception (ρ = 0.318, *p* = 0.006). No significant correlations were found between the levels of psychological distress and TNM stage, or between the levels of psychological distress and adjuvant therapy.

Table 2 shows the mean levels of mood states (POMS) in the MSI-positive group and MSI-negative group at T1 and T2. All mean affective states were within the same range as those observed in other patients diagnosed with cancer [29]. No significant differences were found between the MSI-positive group and MSI-negative group.

### Cancer-specific distress

At T1, cancer-specific distress levels in the MSI-positive group and MSI-negative group were within the same range (Table 2). Mean scores of cancer-specific distress in the study sample were moderate at T1 (21 ± 15) and T2 (21 ± 17). Results of the GLM for repeated measures analysis showed that over time, there were no significant differences in cancer-specific distress levels between the two groups. At T1, 38% of the total group reported high cancer-specific distress (IES ≥ 26); the separate rates were 39 and 38% in the MSI-positive group and MSI-negative group, respectively. At T1 and T2, cancer-specific distress rates in the MSI-positive group and MSI-negative group were 39 versus 27% (McNemar test, exact *p* = 0.3) and 38 versus 36% (McNemar test, exact *p* = 1.0), respectively. At T1, cancer-specific distress scores were significantly correlated with female gender (ρ = 0.328, *p* = 0.005). No significant correlations were found between the cancer-specific distress levels and TNM stage, or between the cancer-specific distress levels and adjuvant therapy.

### Social support and cancer risk perception

At T1 and T2, mean social support levels in the MSI-positive group and MSI-negative group were moderate compared to a norm group of healthy adults [33]. Additionally, there were no significant differences in social support levels between the two groups (Table 2). Table 2 shows that at T1, patients in the two groups reported a fairly high-risk perception of being rediagnosed with CRC in the near future. At T2, risk perception had increased significantly in the total group from 43 to 50% (*t* = 2.237; *p* = 0.03); the separate rates of increase were 43–48% (*t* = 1.409; *p* = 0.2) in the MSI-negative group, versus 44–53% (*t* = 1.948; *p* = 0.07) in the MSI-positive group.

### Partner’s reaction to providing care and support for the cancer patient

Results of the GLM for repeated measures analysis showed significant time effects in the CRA-D and EDIZ questionnaires completed by the partner (F(1, 48) = 7.00, *p* = 0.01 and F(1, 46) = 4.61, *p* = 0.04, respectively). This indicated that the negative impact of providing care decreased in the MSI-positive group and MSI-negative group (Fig. 1b). The partners’ self-esteem (CRA-D) was within the same range as that in the partners of patients with other types of cancer [39] (Table 2). Distress caused by providing informal care (EDIZ) was reported by 49 and 38% of the partners at T1 and T2, respectively. No significant differences in self-esteem and distress were found between the MSI-positive group and MSI-negative group. No significant correlation was found between the partner’s gender and the reaction to providing care.

### Advantages of the MIPA procedure

The MIPA procedure greatly enhances the efficiency of genetic counselling, because there is an increased risk that MSI-high CRC patients are carriers of the mismatch repair (MMR) gene mutation. In our group of 22 MSI-high CRC patients (45%), ten were subsequently found to carry a mutation in one of the MMR genes (n = 6 *MLH1*, n = 2 *MSH6* and n = 2 *PMS2*). In 6 of these patients (27%), MSI was explained by non-hereditary hypermethylation of the *MLH1* promoter. The DNA test result at T2 was not significantly correlated with psychological distress or with cancer-specific distress.

## Discussion

To our knowledge, this is the first multicentre study on psychological distress in patients recently diagnosed with CRC following genetic pre-screening for Lynch syndrome by MSI testing. Our data indicated that disclosure of the MSI test result was not followed by high levels of distress in the majority of these patients. This is in agreement with a previous study in which a shorter time interval between the cancer diagnosis and genetic pre-screening for Lynch syndrome was not related to higher psychological distress [40]. Data from our patients in the MIPAPS pilot study showed that the advantages of early screening, e.g. timely medico-prevention strategies for their children, outweighed any possible disadvantages [41]. These two studies point in the same direction, namely that pre-screening for Lynch syndrome by MSI testing in patients recently diagnosed with CRC is justifiable from a psychological point of view.

Distress and cancer-specific distress levels were moderate in the MSI-positive group and MSI-negative group. However, it is important to note that a minority of our patients with CRC did report high levels of general psychological distress and cancer-specific distress after MSI testing. These high levels of distress decreased over time in the MSI-positive group and remained stable in the MSI-negative group. Six months after MSI test result disclosure, i.e. almost a year after CRC diagnosis, about 20% of the CRC patients were still highly distressed and about 40% were still experiencing high cancer-specific distress. Although the levels of general psychological distress and cancer-specific distress were independent of the MSI test result, they were found to be related to female gender. General psychological distress was also related to low social support and high cancer risk perception. In our study, the overall prevalence of high general psychological distress was lower than that in a previous study in which 32% of the newly diagnosed patients reported high distress [27]. The literature has shown that two-thirds of patients with cancer will adapt to their diagnosis without any psychological intervention [27]. Initial psychological adaptation to the diagnosis of cancer is strongly influenced by pre-existing psychosocial factors [42]. These results highlight the necessity to identify patients with high levels of distress. In our opinion, psychological screening and if indicated, subsequent professional support, should take place soon after CRC diagnosis to avoid or reduce long-term distress.

The results of our study on young patients recently diagnosed with CRC are in line with those from studies on patients recently diagnosed with breast cancer who were actively approached for genetic counselling and testing. Overall, no additional short-term or long-term psychological distress was found in this group of patients [43, 44]. One of the explanations given previously was that the possibly hereditary nature of cancer is not nearly as distressing as the diagnosis of cancer itself [45]. According to our clinical observations, a genetic diagnosis may help patients to understand at least a part of the origin of CRC and reduce psychological distress. However, these observations need to be confirmed by further research.

Another explanation might be that in general, MSI-positive CRC patients have good overall prognoses and there is often less need for adjuvant therapy, as was the case in our study. Therefore, patients who have a high risk of Lynch syndrome may have psychologically compensated for any potentially negative effects based on these factors. The reason why levels of distress and cancer-specific distress remained stable over time in the MSI-negative CRC patients might lie in their poorer prognoses and more general need for adjuvant therapy. However, we could not detect any correlation between adjuvant therapy and psychological distress.

The partners of the patients in the MSI-positive group and MSI-negative group showed moderate to high levels of self-esteem. These levels were comparable with those described in the literature on partners of patients with CRC [35] or other types of cancer [39]. In the two groups, levels of perceived distress decreased over time. This was in concordance with the previous literature in which the treatment phase was experienced as the most stressful period, as it involved the greatest need for emotional and informational support [46].

One limitation of our study was the low response rate in the eligible patients. This may have biased our results, especially if the surgeons had consciously avoided recruiting patients with a (very) poor prognosis or emotional problems. Such bias would have resulted in underestimation of psychological distress. At present, we cannot assess whether bias was present. However, we note that in our sample, the levels of psychological distress were lower than those described in the literature. Another reason for the low response rate may have been the complex logistic inclusion procedure [15], if communication of the test result to the patient exceeded the inclusion criterion of 6 months. In some cases, it took several months before the MSI-test report, written by the pathologist, was sent to the surgeon and a number of weeks more before the patient was contacted. Another limitation of our study was that no firm conclusions could be drawn, because the large number of tests increased the possibility of a type I error, which we have not corrected for.

Despite some methodological concerns, we can conclude that moderate levels of distress were present following MSI testing in patients recently diagnosed with CRC. These levels were similar to those in other patients diagnosed with CRC [27, 47, 48]. High cancer-specific distress was observed in 40% of the MSI-positive patients and was significantly correlated with female gender.
